# Supplementary material for: Proteogenomics of Colorectal Cancer Liver Metastases: Complementing Precision Oncology with Phenotypic Data
Source: Cancers (Basel). 2019 Dec 1;11(12):1907. doi: 10.3390/cancers11121907 (PMC6966481; doi:10.3390/cancers11121907)
Supplement: Supplementary file 1 [file cancers-11-01907-s001.zip › cancers-645556-suppl-XML/cancers-645556-suppl-Figure.docx]

Supplementary Materials: Proteogenomics of Colorectal Cancer Liver Metastases: Complementing Precision Oncology with Phenotypic Data

Bernhard Blank-Landeshammer , Vincent R. Richard, Georgia Mitsa, Maud Marques, André LeBlanc, Laxmikanth Kollipara, Ingo Feldmann, Mathilde Couetoux du Tertre, Karen Gambaro,
Suzan McNamara, Alan Spatz, René P. Zahedi, Albert Sickmann, Gerald Batist and
Christoph H. Borchers


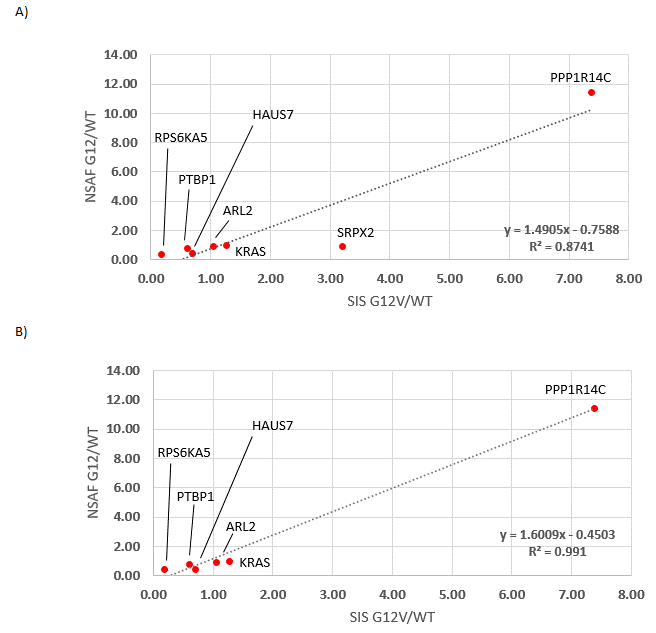


**Figure S1.** Good correlation between SIS-peptide and NSAF-based G12V/WT protein ratios. G12V/WT ratios were determined based on NSAF (Y-axis) and absolute quantification using SIS peptides (X-axis). (**A**) Plotting both ratios shows a good correlation, however, SRPX2 is a clear outlier. (**B**) Removal of the outlier SRPX2 leads to an R^2^ of 0.99.

| 1. 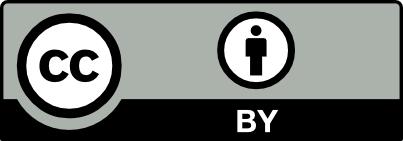 | 1. © 2019 by the authors. Licensee MDPI, Basel, Switzerland. This article is an open access article distributed under the terms and conditions of the Creative Commons Attribution (CC BY) license (http://creativecommons.org/licenses/by/4.0/). |
| --- | --- |
